# Extending XNAT Platform with an Incremental Semantic Framework

**DOI:** 10.3389/fninf.2017.00057

**Published:** 2017-08-31

**Authors:** Santiago Timón, Mariano Rincón, Rafael Martínez-Tomás

**Affiliations:** ^1^Departamento de Inteligencia Artificial, Universidad Nacional de Educación a Distancia Madrid, Spain; ^2^Department of Neurology, Akershus University Hospital Lørenskog, Norway; ^3^Intervention Centre, Oslo University Hospital Oslo, Norway

**Keywords:** biomedical ontologies, Semantic Web, knowledge management, XNAT, data exchange, data analysis, Neurodegenerative Diseases

## Abstract

Informatics increases the yield from neuroscience due to improved data. Data sharing and accessibility enable joint efforts between different research groups, as well as replication studies, pivotal for progress in the field. Research data archiving solutions are evolving rapidly to address these necessities, however, distributed data integration is still difficult because of the need of explicit agreements for disparate data models. To address these problems, ontologies are widely used in biomedical research to obtain common vocabularies and logical descriptions, but its application may suffer from scalability issues, domain bias, and loss of low-level data access. With the aim of improving the application of semantic models in biobanking systems, an incremental semantic framework that takes advantage of the latest advances in biomedical ontologies and the XNAT platform is designed and implemented. We follow a layered architecture that allows the alignment of multi-domain biomedical ontologies to manage data at different levels of abstraction. To illustrate this approach, the development is integrated in the JPND (EU Joint Program for Neurodegenerative Disease) APGeM project, focused on finding early biomarkers for Alzheimer's and other dementia related diseases.

## Introduction

Nowadays, neuroscience research projects take place in multidisciplinary, heterogeneous multi-center environments, where an efficient mean of data exchange is crucial. One of the main challenges is the accurate and effective exchange of data for its subsequent analysis, that leads to the need of a common structure, data standardization or some mediation strategies (Ashish et al., 2010). Some currently in use archiving solutions, as reviewed in Izzo (2016), are the Extensible Neuroimaging Archive Toolkit (XNAT) (Marcus et al., 2007), the Collaborative Informatics and Neuroimaging Suite (COINS) project (Scott et al., 2011), or the eXTENsible platform for biomedical Science (XTENS) (Corradi et al., 2009).

Despite the flexibility and ease of customization offered by the mentioned archiving systems, data scalability is somehow limited, as significant changes in the data model typically require fine configuration of the database or an important reorganization. These shortcomings have been addressed by the use of ontologies and Semantic Web technologies (mainly OWL^1^, RDF^2^, and SPARQL^3^) (Hoehndorf et al., 2015). The Mayo Clinic made one of the first examples of such approach by applying Linked Data principles to its Electronic Health Records (Pathak et al., 2012a). They leveraged publicly available data from the Linked Open Drug Data cloud (Samwald et al., 2011) to federated querying for type 2 diabetes patients. Following the same principle, Leroux and Lefort (2015) showed an efficient approach to enrich the semantics in clinical trials. They developed a semantic, linked data model from CDISC Operational Data Model^4^, focusing just on the easy data sharing and consumption, and leaving further modeling and reasoning for the future. On a more domain-specific context, Hsu et al. designed an ontology-driven system employing an application ontology that imports and aligns ontologies from different domains (Hsu et al., 2015). It integrates phenotypes generated through analyses of available clinical data sources. Their approach demonstrated how an ontological framework could help to enforce consistent data representation and even enable further studies to identify clinical predictors. Also, numerous approaches have been proposed for complex knowledge intensive tasks in the past years, like radiological assistance (Mejino et al., 2008), surgical planning (Mechouche et al., 2007, 2009), or clinical management (Sonntag, 2008) and patient care systems (Su and Peng, 2012).

Notwithstanding the obvious growth in its application, the adoption of ontological frameworks shows some drawbacks and is still a challenging and time consuming venture (Hastings et al., 2014). There exists a trade-off between the language expressiveness and its computational tractability that requires making decisions about the necessary level of description. Usually, the use of highly descriptive ontologies alone results in *ad-hoc* implementations for domain-specific solutions with poor scalability that complicates raw data extraction for less knowledge-aware tasks. Furthermore, ontology selection, alignment, and mapping require the collaboration of domain experts and development staff, in addition to the steep learning curve for new users of ontologies. Ontology engineering methodologies, such as the NeOn Methodology (Suárez-Figueroa et al., 2012) provide a methodological guide for addressing several of the mentioned issues, usually targeted at a final high-level ontological ecosystem. However, leaving behind intermediate low-level data is problematic when the goal is integrating complex, distributed systems. The loss of the original data structure compromises data quality and limits the possibilities for its manipulation at the same time. A Bottom-up approach that supports all description levels simultaneously is more convenient for these projects. It has been successfully applied in other domains, for e.g., in the video analysis domain (Duan et al., 2003).

In this article, we describe an incremental semantic framework; a methodological approach to address the problem of enabling semantic-based modeling in already implemented research archiving systems. Consequently improving data management, from low-level data to semantic and logical concepts. Built with Semantic Web technologies and using biomedical ontologies, the framework provides a model for homogenous data access and reasoning over multi-modal neurological data.

The design of the framework follows a bottom-up, layered approach, allowing working with the data at different levels of description. The framework adds reasoning capabilities from implicit relations and logical definitions to derive new data, as well as to perform data consistency checks for Quality Control (QC). The use of Linked Data principles enables inter-data linking, opening the door to reference external data sets. Also, having a highly linked dataset eases data inspection from different conceptualizations (project, subject, disease, etc.), a highly desirable feature for pattern discovery and studying the relationship between diseases as the dataset grows.

Our proposal differs from previous works in its focus on advanced querying and reasoning without losing low-level data, while taking advantage of already available and widely used archiving platforms. Particularly, we chose XNAT as the backbone for managing clinical and imaging data, for its rich set of features and its flexible and customizable design.

To illustrate the benefits of the framework, this work is encompassed in the JPND (EU Joint Program for Neurodegenerative Disease)^5^/APGeM project^6^, aimed at finding early biomarkers for Alzheimer's and other dementia related diseases. It comprises a significant amount of data from different subdomains and modalities, such as neuroimaging, biochemistry, clinical/neuropsychological screenings and genetics, setting up a proper scenario to push and test the framework with a current ongoing neurological research effort.

The remainder of the paper is organized as follows. In Section Material and Methods we describe the design and technological methodology, as well as the data from APGeM's project. Next we exemplify the utility of the framework through various use case applications in Section Results. Finally, in Section Discussion we discuss the benefits, problems encountered and limitations of our implementation and conclude in Section Conclusion.

## Materials and methods

This section starts describing the data from the APGeM project. It is part of the driving material and an example of application of the semantic framework. Later, in Section Data Management with XNAT Platform we describe the features of the XNAT platform. In Section Framework Design we outline the decisions made to design each layer of the ontological framework. Finally, in section Data Transformation and Storage, we describe the details of the transformation and loading of the data for persistence.

The related code that is not core to APGeM is available at https://bitbucket.org/apgem-isf/ under Apache Licence, version 2.0.

### APGeM project data

The APGeM project, where this work is encompassed, is focused on finding early biomarkers for Alzheimer's and other dementia related diseases (Fladby et al., 2017). It comprises individuals assessed with subjective cognitive decline (SCD) (Jessen et al., 2014), mild cognitive impairment (MCI) (Albert et al., 2011), dementia, and healthy controls.

Subjects were recruited from January 2013 to January 2017 and examined following a standardized protocol. Recruitment was based on two main sources: (1) self-referred patients following advertisements in media, newspapers, or news bulletins, and (2) recruited patients among referrals to regional memory clinics. In addition, cognitively healthy controls were also included from spouses of patients with dementia/cognitive disorder, and from patients who completed lumbar puncture for orthopedic surgery. Participants were staged as controls, SCD or MCI using published criteria based on the comprehensive assessment program. Controls were further classified as having normal or abnormal cognitive screening and with or without first-degree relative with dementia.

A case report form (CRF) was developed, comprising medical history (captured from subject and informant separately), and physical and neurological examinations including the 15-item Geriatric Depression Score (Mitchell et al., 2010). The cognitive examination included the Mini Mental State Examination (Folstein et al., 1975), non-verbal cognitive screening (The clock drawing test) (Shulman, 2000), verbal memory (Fillenbaum et al., 2008), visuoperceptual ability, psychomotor speed, and divided attention (Trail making A and B and word fluency). The dataset also included relevant biomarkers for Alzheimer's and other dementia related diseases, obtained from Cerebrospinal fluid and blood samples.

All subjects were referred to a standardized magnetic resonance imaging (MRI) scan protocol; including high resolution structural scans. A sub-set of subjects also underwent an extended MRI protocol including advanced diffusion weighted sequences as well as multiple positron emission tomography (PET) modalities.

### Data management with XNAT platform

The Extensible Neuroimaging Archive Toolkit (XNAT—RRID:SCR_003048) is an archiving software platform designed to facilitate common management and processing tasks for neuroimaging and related data, providing a secure storage and access layer. XNAT's architecture follows a three-tier design pattern that includes a relational database backend, Java-based middleware engine, and a web-based user interface.

The key of XNAT's flexibility resides in the XML-based data model that defines the data-types that are to be handled by the deployed system. XNAT uses these XML schemas^7^ (XSD) to generate custom components, content, and logic for each of the tiers: (1) a relational database structure is generated, equivalent to the elements defined in the XSDs; (2) middleware classes are generated that can be used by developers to implement custom functionality that utilizes the XNAT database; and (3) user interface content, including navigation menus, search options, and data tables. This building mechanism allows research groups to customize data-types and interfaces for storing the relevant data to their studies. The level of this customization is left to developers, going from implementing simple types and questionnaires to complex data structures, interactive interfaces, and business logic.

Another fundamental part is the REST (Fielding and Taylor, 2002) API. It allows interacting with XNAT through HTTP protocol to support basic actions like Create, Read, Update, and Delete resources, as well as more advanced features like data searching and listing, which permits to integrate external pieces of software with XNAT.

Finally, XNAT also ships a pipeline engine that tightly integrates and manages processing pipelines into XNAT's workflow. This was another key feature for the platform selection process, since pipeline execution is critical in Neuroimaging research to develop tasks such as image quality control and automated segmentation.

To this day, there are several publicly available solutions to manage clinical and omics data more efficiently than XNAT, such as BRISK, caTRIP, cBio Cancer Portal, G-DOC, iCOD, iDASH, and tranSMART (Scheufele et al., 2014; Canuel et al., 2015), existing the option to implement a distributed data warehouse system and leave XNAT in charge of neuroimaging data. However, while adapting and customizing XNAT to fit the project needs was a time consuming task, the learning curve was applied only to one system. This allowed for better understanding and, consequently, maximizing the exploitation of XNAT's features.

### Framework design

**Figure 1:**
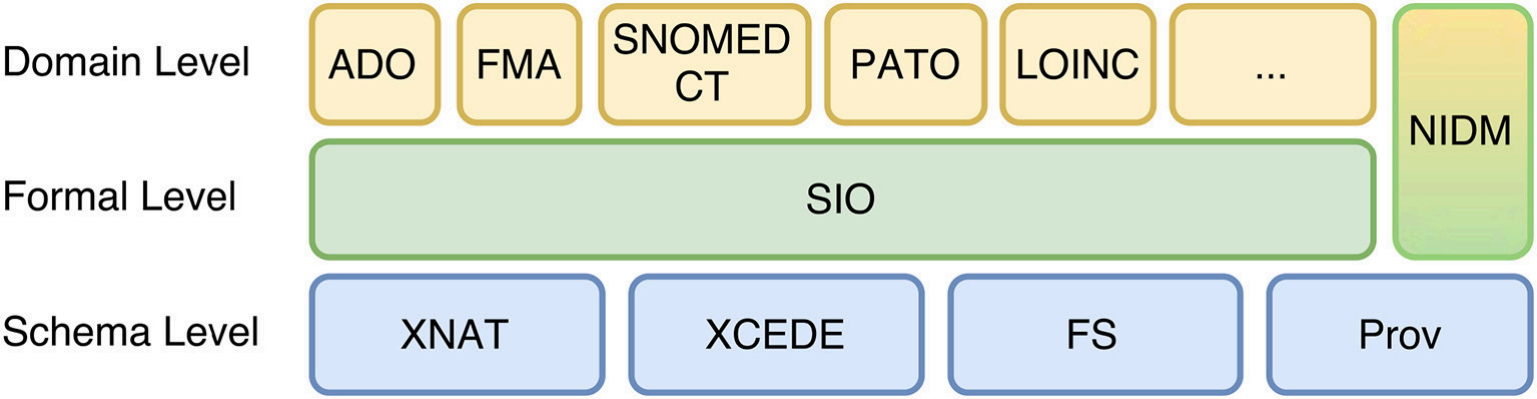
The three component layers of the framework, ordered by abstraction.

Conceptually, the framework follows an n-tiered incremental design, composed of three layers, or levels (Figure 1): schema, formal and domain. This approach intends to add the complexity cumulatively, in a way that is possible to access low-level data easily (schema and formal levels) and look for further relations and descriptions based on logical axioms at the same time (formal and domain levels). The schemas and ontology acronyms included in Figure 1 are described in related subsections.

The schema level is the entry point of the framework; it defines the source data structure through XML schemas. The formal level delivers the data modeled with vocabularies under Semantic Web standards. It augments the basic semantics of the Schema level introducing more abstract concepts. These concepts are defined through Description Logics and translated to a RDF graph model without losing completely its source, which allows low-level inspection and data retrieval and also introduces more refined provenance descriptions. Finally, the domain level provides more expressive descriptions to enable further reasoning and query capabilities, for instance, using richer domain specific ontologies to include neuroanatomical terms and mereological axioms.

#### Schema level

The core data model of XNAT supports the storage of imaging and custom clinical data, laying the foundation for the schema level, the first layer of the semantic framework. XNAT itself models the basic organizational and imaging data structures, leaving further extensions for other three schemas used in this layer, XCEDE, FreeSurfer (FS) (FreeSurfer, RRID:SCR_001847) and W3C Provenance data model^8^.

While XNAT schema is well fitted for data persistence, its expressivity is somehow limited for describing the study design. We use the XCEDE (XML-based Clinical and Experimental Data Exchange) schema (Gadde et al., 2012) (XCEDE Schema, RRID:SCR_002571) to keep the imaging part of the CRF and describe the study and protocol design under the same specification. The existing overlap between XNAT and XCEDE models facilitates mapping data in both ways and complements the core data model of XNAT.

We leave XNAT schema to focus on data persistence and, as a previous step before introducing more descriptive semantics, employ XCEDE to describe the study protocol in an exchangeable format and link to ontology terms from upper levels in the framework through the “Terminology” component of the schema.

To integrate XCEDE import/export processes properly, we have implemented an XNAT service extension following the same principles as its native REST API to serve study data in XCEDE format. The service serves data by employing several transformation scenarios designed for each resource type defined in the model.

The XNAT community provides the FreeSurfer schema, enabling a means to store FreeSurfer results into XNAT and share them between researchers. Furthermore, having a results XML model eases its processing at higher levels in the framework.

The schema level makes possible to work with XNAT's native data format for low-level data processing, while enabling at the same time data sharing and further modeling through less platform specific schemas. This is very valuable in situations where low-level inspection is needed and abstractions are not beneficial or even counterproductive.

#### Formal level

The formal level provides an entry level to model the data through Semantic Web technologies. It serves as the foundational layer to model XNAT experiment data as information entities that describe data, studies and protocols, and which could be further aligned or mapped to specific domain ontologies. It improves low-level semantics by introducing logical definitions with Description Logics (DL), more powerful sharing mechanisms with data linking, query strategies, and finally enabling DL reasoning.

We used NCBO's Bioportal (Musen and Noy, 2011; Whetzel et al., 2011) (BioPortal, RRID:SCR_002713) to find the most suitable ontology. After evaluating various ontologies based on the Basic Formal Ontology^9^ (BFO, RRID:SCR_004818) upper-level model, such us the Ontology of Clinical Research (OCRe) (Sim et al., 2014) (Ontology of Clinical Research, RRID:SCR_010392), the Translational Medicine Ontology (TMO) (Luciano et al., 2011), the Semanticscience Integrated Ontology (SIO) (Dumontier et al., 2014) (Semanticscience Integrated Ontology, RRID:SCR_010427), and the Neuroimaging Data Model (NIDM)^10^ (Keator et al., 2013) (Neuroimaging Data Model, RRID:SCR_013667), we concluded that SIO covers more terms related to low-level information representation in contrast with OCRe. Also, SIO can be seen as the supported successor of TMO, as it emerged from considerations in the TMO effort. Finally, NIDM is less formal than SIO, but models in more detail concepts related to neuroimaging. On this basis, we decided to employ an alignment of SIO and NIDM as the foundational ontologies to model CRF and imaging data. On the one hand, SIO was used to describe studies and protocols and also to model information entities and experiment data. On the other, NIDM was used to model important provenance and processing neuroimaging results data (Maumet et al., 2016).

At this level, the core elements in the base XNAT data model had to be properly mapped to concepts of SIO. For versions 1.6.x, these elements were Project, Subjects, and Experiments, and some of them lack of direct correspondence with SIO. Most of the mapping process is as detailed bellow.

The term “experiment” in the SIO ontology is defined as an “investigation that has the goal of verifying, falsifying, or establishing the validity of a hypothesis,” while for XNAT it is an event by which data is acquired. Therefore, the meaning for “experiment” differs between them and we found “data collection” a suitable entity to model experiment data in XNAT's sense, encoding final literal data with “data item” instances. The description for the entity “data collection” is defined as the process of acquiring information. Adding the insertion/collection date to “data collection” instances complies with XNAT definition of experiment. Hence, the basic starting point to model experiment data is using Data collection class for experiment instances, which has output sub-sections as data set instances. These specify the data fields with has data item property and data item instances. The final values are literals related with has value data type property. Formally in DL notation:

$$\begin{array}{l} Data\;collection⊓(\exists has\;output.(Data\;set\\ \;⊓(\exists has\;data\;item.(Data\;item))) \end{array}$$

**Figure 2:**
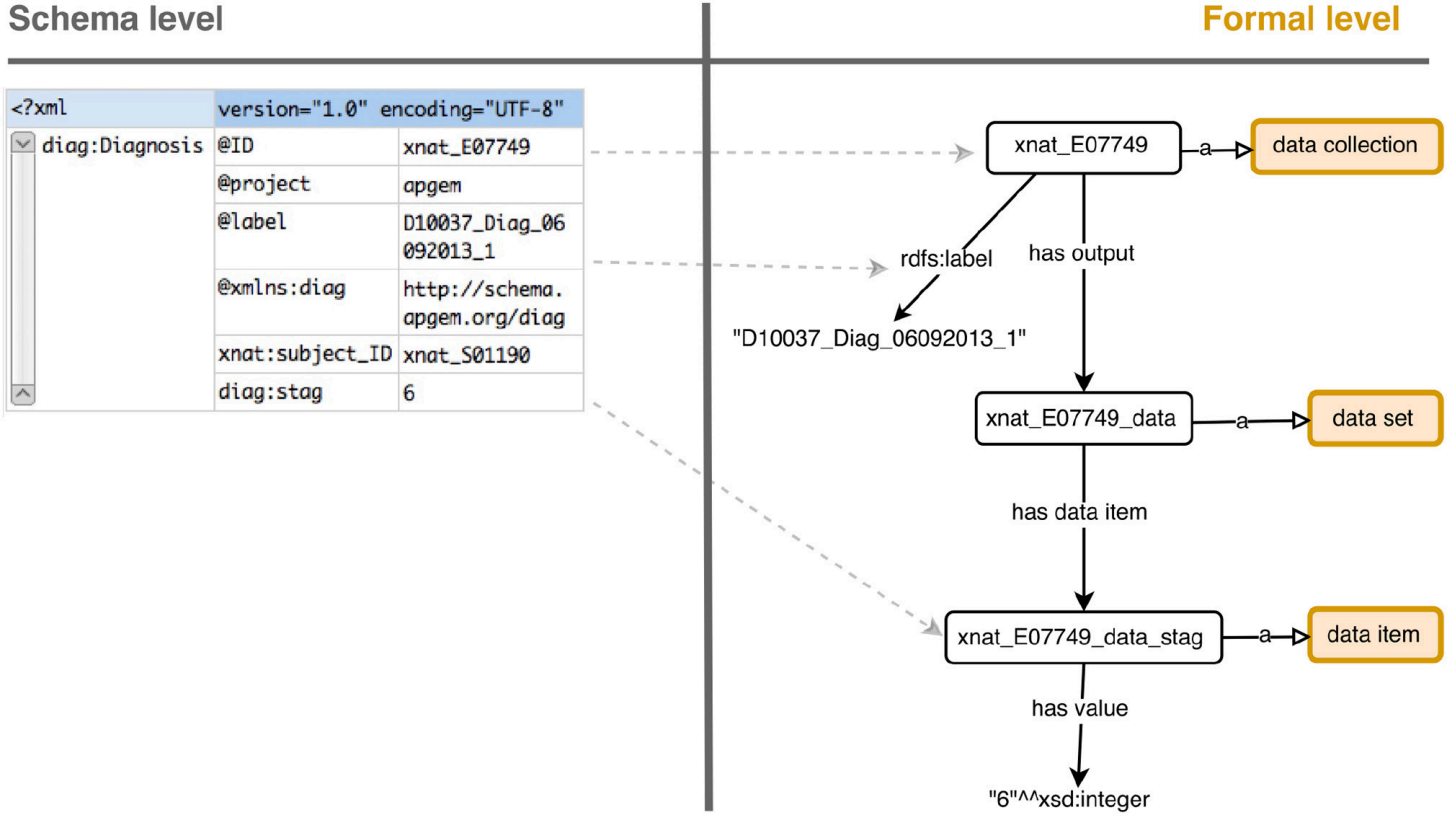
Schema to formal level transformation from a stripped down experiment for exemplification. XNAT XML data is transformed to an RDF graph using SIO classes and properties. Instances are represented as white rectangles and classes as rounded orange rectangles.

Figure 2 depicts the basic means to represent an experiment and its data. It is important to note that, depending on the experiment type, the way of obtaining raw values may differ and should be consequently modeled, distinguishing between observations (a doctor's assessment), measurements with values and units (the amount of blood cholesterol) or test outputs (the T-Score for TMT test).

#### Domain level

Up to this level, the meaning of the data elements is still kept at low level, leaving the interpretation to *ad-hoc* processes or humans from coding conventions. The purpose of the domain level is to provide high-level semantics and, when possible, logical definitions for the concepts depicted in the data and even rules to further enrich the model. This level tends to be specific to the application or context of the project, thus the ontology selection and modeling decisions depend heavily on it. We demonstrate the building of this level through its application to the Alzheimer's Disease domain.

The Alzheimer's disease ontology (ADO) (Malhotra et al., 2014) (ADO, RRID:SCR_010289) is the first bridge for our use case domain context, focused in Alzheimer's and related diseases. ADO was developed with the purpose of containing information relevant to four main biological views: preclinical, clinical, etiological, and molecular/cellular mechanisms, making possible to map and classify most of the CRF items from APGeM project. The SNOMED CT (Cote and Robboy, 1980) ontology is widely adopted because of its comprehensive clinical terminology. It was used to cover many of the leaf clinical terms in almost every experiment type. To reference anatomical entities we selected the Foundational Model of Anatomy (FMA) (Rosse and Mejino, 2003) (FMA, RRID:SCR_003379) because of its completeness and robust representation of the anatomical reality (Zhang et al., 2003). The Phenotype And Trait Ontology (PATO)^11^ was employed to represent biological and phenotypic qualities. The Logical Observation Identifiers Names and Codes (LOINC)^12^ (Huff et al., 1998; McDonald et al., 2003) (Logical Observation Identifier Names and Codes, RRID:SCR_010341) was a suitable terminology to map biochemical tests (Bakken et al., 2000), complemented with SNOMED terms. Finally, genetics were mapped to Gene Ontology concepts (Ashburner et al., 2000; Gene Ontology Consortium, 2010). Table 1 shows a summary of the application of the ontologies to the different sub-domains.

**Table 1:** Relation of the component parts of the CRF with subsections and the ontologies with which are modeled.

**CRF experiment/questionnaire**	**Subsections**	**Ontology/Vocabulary**
Subject demographics		PATO SNOMED CT
Medical history	Social information Family history Current medical history (participant and informant) Current medication Stimulants Other bodily functions Previous medical history Geriatric depression scale	ADO SNOMED CT Disease Ontology
Cognitive screening	MMSE CERAD word list Trail Making Test COWAT (FAS) VOSP silhouettes Clinical dementia rating	ADO SNOMED CT Disease Ontology
Physical examination	General somatic examination Neurological Exam UPDRS Modified UPDRS	ADO SNOMED CT FMA
Diagnosis	Staging Etiology	ADO SNOMED CT Disease Ontology
Biochemistry	Blood tests Spinal puncture (CSF)	ADO LOINC Snomed CT
Genetics		ADO Gene Ontology
Imaging reports		NIDM ADO SNOMED CT FMA

In a typical research project, each experiment type introduces a significant amount of variables (more than 1,100 categorized across several sub-domains in our use case) that need to be mapped to concepts from domain ontologies, implying a very time consuming task. To assist and reduce the time needed in the process of finding term candidates, we developed a script that uses XNAT's search engine through PyXNAT library (Schwartz et al., 2012) (pyxnat, RRID:SCR_002574). For each data-type schema, it inspects complex and simple types to extract the variables to be mapped. Then, for each variable a query is sent to Bioportal's search endpoint with a list of candidate ontologies. The response is a collection of candidate terms for the variable, among other related information, such as the ontology in which the term is defined. The output is an XML file with possible term mappings for each variable. This process has saved a fair amount of time and resources for the ontology and concept selection.

**Figure 3:**
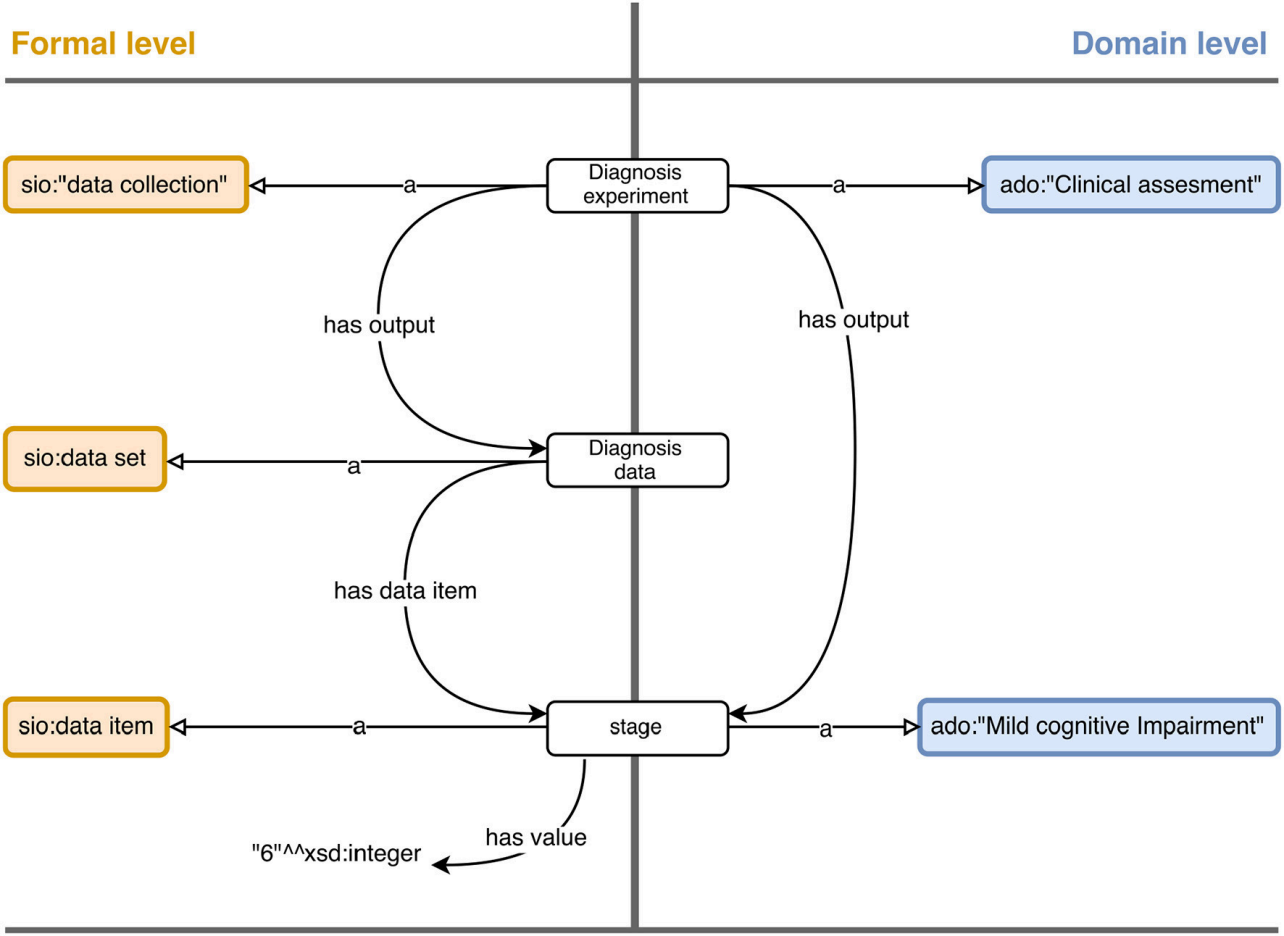
A diagram showing the relations building the assertion that a subject is staged with Mild Cognitive Impairment. The formal level improves the semantics of experiment data, but is still attached to raw values. The domain level introduces specific concepts for a given domain, in this case diagnosis in Alzheimer's Disease. Instances are represented as rounded white rectangles and classes as rounded colored rectangles.

Figure 3 shows an example of mappings at formal and domain level.

The domain level for the project was built through the alignment of the selected ontologies. We imported them when possible and, for those too big or broad to be imported, we followed the MIREOT process (Courtot et al., 2011) to include terms of interest. Finally, further logical restrictions and rules relevant to the domain of the use case were defined.

### Data transformation and storage

At the schema level, the mappings were almost direct between XNAT data model and XCEDE. The transformation was accomplished with XSLT^13^ (eXtensible Stylesheet Language Transformations), served on the fly over XNAT's API endpoint. However, before entering the semantic framework, XNAT source data was transformed and mapped to the target model.

**Figure 4:**
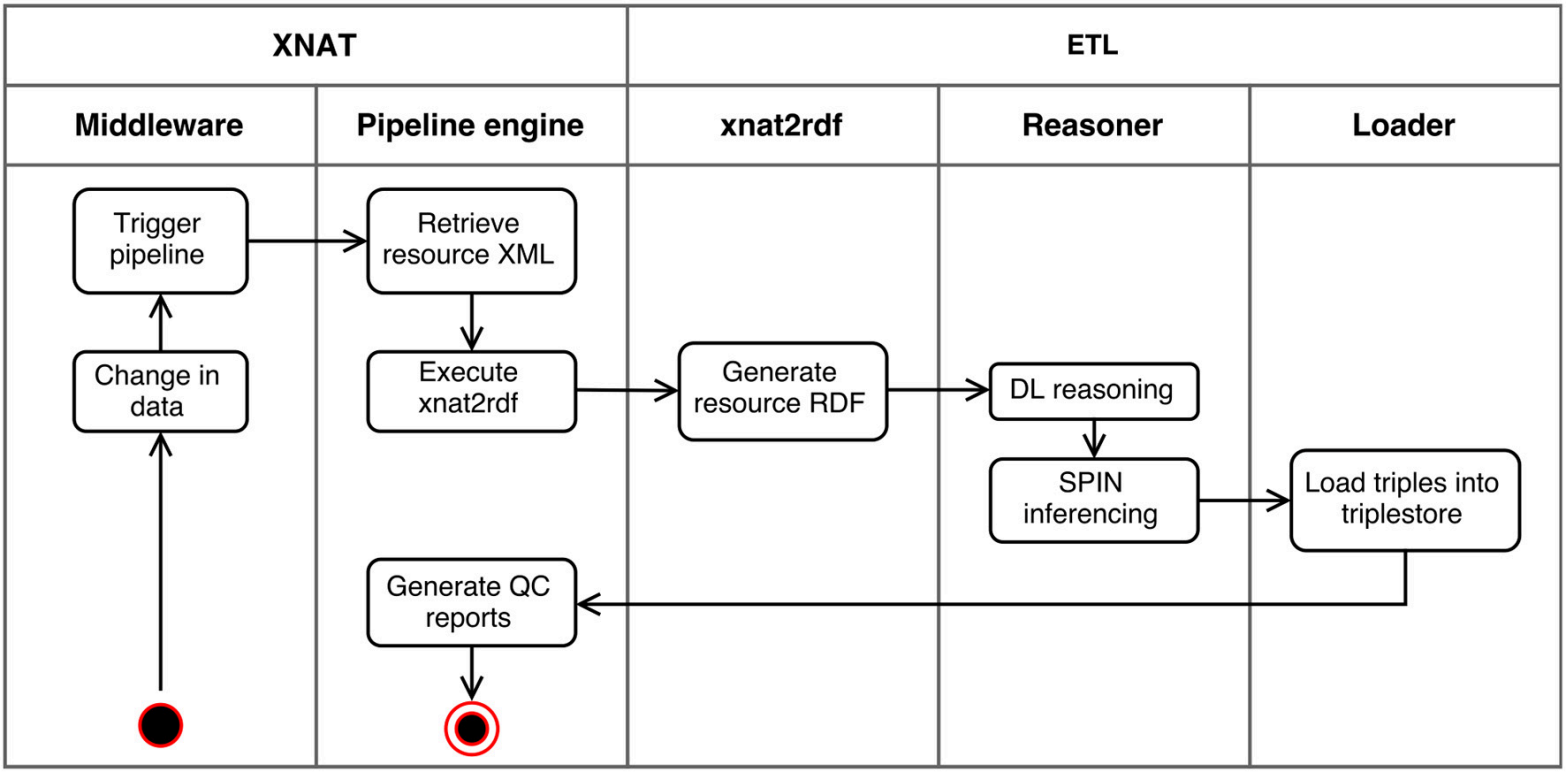
Activity diagram of the ETL pipeline. When any change in the data is registered by XNAT's middleware, the pipeline engine executes the xnat2rdf script passing the XML of the changed resource. This script transforms XNAT XML to RDF, which is processed by the reasoner to execute DL and SPIN inferencing and the resulting triples loaded into the triplestore. Finally QC related data is processed for reporting.

To expose subject and experiment data coming from XNAT as RDF, the Extract-Transform-Load (ETL) pipeline depicted in Figure 4 was implemented.

The workflow is as follows: when any update operation is performed in XNAT the pipeline retrieves the XNAT resource XML and, executes the xnat2RDF script, which transforms it to RDF format using both formal and domain level models. These generated triples are then processed for reasoning, using Pellet reasoner (Sirin et al., 2007) and SPIN^14^ (SPARQL Inferencing Notation) API. The output triples from the reasoner script are then loaded into a Jena (JENA: A Semantic Web Framework for Java, RRID:SCR_001766) Fuseki 2^15^ triplestore instance.

The primary criterion for the selection of technologies was the ease of integration between the different parts of the workflow, in spite of sacrificing efficiency in some of the steps. Because the execution of this transformation process is made “offline,” its performance is not critical to the system's usage. Nevertheless, the execution time is restrained, lasting a couple of seconds per complete subject data (demographics and all experiment data included in the CRF), and less than one second for individual resources.

Fuseki SPARQL Server performs very well in most of the triplestore related operations (Butt and Khan, 2014), although it suffers from write performance problems (Kilintzis and Beredimas, 2014). The reasoning step can be tuned and adapted to use different OWL profiles to reduce execution time. It would be also beneficial to use high-performance reasoning engines like Konclude (Steigmiller et al., 2014), the winner of OWL Reasoner Evaluation 2015 (Parsia et al., 2017). However, these changes would turn into a slightly more complex setup for the ETL process.

We followed the recommendations from the Interoperability Solutions for European Public Administrations (ISA^2^) for the design of persistent URIs^16^ that represent the generated resources (instances).

## Results

To illustrate the utility of the proposed design methodology, our framework was integrated into the system environment of APGeM. In order to ensure secure access to sensitive medical data, the environment runs on the Services for sensitive data (TSD) provided by the University of Oslo.

The following sections describe how the integration of the framework enabled data science researchers to engage QC, subject classification, and advanced reporting tasks through semantic querying and logical reasoning.

### Data quality control

Nowadays, the data managed in neuroscience research projects cover very different biomedical fields and is therefore gathered by several, diverse means, such as laboratory reports for biochemical tests, interviews for screening data, MRI acquisitions, and so on. The data obtained is then entered into XNAT by human collaborators or semi automated processes that need human interaction at some point of their workflow, which is prone to introduce errors and inconsistencies in the dataset. Having a sound, error free, dataset is crucial for any data analysis process. Consequently, there is a need for designing a QC strategy that effectively detects and manages this kind of errors. To tackle the QC problem our approach is based on ontology-based data quality management principles. It takes advantage of the logical model defined in the ontologies and expands it with more explicit SPIN rules and constructs.

After transformation, the reasoning step of the ETL pipeline derives data and carries out consistency checks. The reasoner checks the logical restrictions defined in the model to assure data consistency. Simultaneously, the definition of constraints using SPIN rules is also valuable for further and more fine-grained inspections that may be difficult to model using Description Logics alone (Fürber and Hepp, 2010).

The layered approach for the semantic model enables working at different levels of abstraction, which allows to verify raw data from XNAT (e.g., assuring the experiments follow predefined ID patterns) and to control more abstract conceptualizations at the same time.

An example of a high-level QC task is finding subjects who meet the exclusion criteria but have not been properly tagged by human supervisors. These errors introduce noise in the data analysis models but are easily overlooked. For this task, ADO defines the class “exclusion criterion,” with a set of specific subclasses modeling several exclusion criteria that covered most of the needs of this project. Depending on which of the variables from the subject's medical history experiment are set to true, the subject is related to the specific instance that represents the exclusion. This check is modeled by the SPIN constraint depicted in Code 1.

Code 1 **SPIN constraint to determine if a subject meets exclusion criteria.**


PREFIX sio: <http://semanticscience.org/resource/>
PREFIX rdfs: <http://www.w3.org/2000/01/rdf-schema#>
PREFIX apgem: <http://www.apgem.org/resource/>
PREFIX snomed: <http://purl.bioontology.org/ontology/SNOMEDCT/>
 
# SPIN reserved word “this” refers to the evaluated instance of
# 'Study subject'
 ASK WHERE {
     # This data is obtained from Medical History experiment, previous and
     # current medical history sections.
     ?this sio:SIO_000062 ?mhExperiment.
     ?mhExperiment a apgem:apgem_0003 ; sio:SIO_000312 ?mhdata.
 
     # The exclusion criteria is met when the subject has filed
     # any of these symptoms:
     # Cerebral infarction, cerebral hemorrhage, epilepsy,
     # head trauma with loss of consciousness,
     # infection in CNS, bipolar disorder, psychosis,
     # delirium/confusion or long term exposure to solvents
     # and malignancy.
     ?mhdata sio:SIO_000028*/sio:SIO_001277 ?cb, ?ch, ?epilepsy, ?ht, ?cnsInfection,
        ?bipolar, ?psychosis, ?delirium, ?exposure.
 
     # each data item 'denotes' the conditions under SNOMED
     # and the item must have 'true' as value
     ?cb sio:SIO_000020 snomed:432504007 ; sio:SIO_000300 true.
     ?ch sio:SIO_000020 snomed:274100004 ; sio:SIO_000300 true.
     ?epilepsy sio:SIO_000020 snomed:84757009 ; sio:SIO_000300 true.
     ?ht sio:SIO_000020 snomed:82271004 ; sio:SIO_000300 true.
     ?cnsInfection sio:SIO_000020 snomed:128117002 ; sio:SIO_000300 true.
     ?bipolar sio:SIO_000020 snomed:13746004 ; sio:SIO_000300 true.
     ?psychosis sio:SIO_000020 snomed:69322001 ; sio:SIO_000300 true.
     ?delirium sio:SIO_000020 snomed:2776000 ; sio:SIO_000300 true.
}

### Automatic staging

A central task within the APGeM project is assessing the subject's stage in cognitive decline for diagnostic purposes and it can be automated based on available screening data stored in XNAT. On the one hand, it is another mean of QC for submitted data, highlighting possible discrepancies between evidence in the screening tests and the final outcome, which may be due to a human error made at data entry or an incorrect diagnosis from the practitioner. On the other hand, it produces useful staging information when the diagnostic interview is missing for any reason. Moreover, the comparison with the manual staging performed by a physician is also noteworthy.

Our approach integrates a simple stage classifier as part of both formal and domain layer. The subject can be staged under 5 different categories, described in Table 2. The classifier has been implemented as a set of SPIN rules (Code 2) that assess the diagnostic staging by filtering screening data that meets several conditions for different clinical tests.

**Table 2:** Description of stage categories and simplified criteria definition with Description Logics.

**Class**	**Description**	**Simplified formal definition**
Normal Control (NC)	The subject's MMSE score is over 28, all T-Scores are equal or greater than 35 and does not report subjective cognitive decline	*Normal* ≡ *StudySubject* ⊓(∃*mmse*. ≥ 28) ⊓(∃*Tscore*_*VOSP*_. ≥ 35) ⊓(∃*TscoreCOWAT*. ≥ 35) ⊓(∃*Tscore*_*CERAD Recall*_. ≥ 35) ⊓(∃*Tscore*_*TMTB*_. ≥ 35)⊓ ¬(∃*reports*.*SCD*)
Subjective Cognitive Decline (SCD)	The subject's MMSE score is over 28, all T-Scores are equal or greater than 35 and reports subjective cognitive decline	*SCD* ≡ *StudySubject* ⊓ (∃*mmse*. ≥ 28) ⊓(∃*Tscore*_*VOSP*_. ≥ 35) ⊓(∃*Tscore*_*COWAT*_. ≥ 35) ⊓(∃*Tscore*_*CERAD Recall*_. ≥ 35) ⊓(∃*Tscore*_*TMTB*_. ≥ 35) ⊓(∃*reports*.*SCD*)
Mild Cognitive Impairment (MCI)	The subject's MMSE score is between 23 and 28, having at least one T-Score under 35	*MCI* ≡ *StudySubject* ⊓ (∃ mmse. > 23) ⊓ (∃*mmse*. < 28) ⊓ ((∃ Tscore_*VOSP*_. < 35) ⊔ (∃*Tscore*_*COWAT*_. < 35) ⊔ (∃*Tscore*_*CERAD Recall*_. < 35) ⊔ (∃*Tscore*_*TMTB*_. < 35))
Dementia	The subject's MMSE score is under 23 and has at least one T-Score under 35	*Dementia* ≡ *StudySubject* ⊓ (∃*mmse*. ≤ 23) ⊓((∃*Tscore*_*VOSP*_. < 35) ⊔(∃*Tscore*_*COWAT*_. < 35) ⊔(∃*Tscore*_*CERAD Recall*_. < 35 )⊔(∃*Tscore*_*TMTB*_. < 35 ))

Code 2 **SPIN rule attached to study subject class instances. It constructs new triples to the subject's diagnosis experiment and state Mild Cognitive Impairment at both formal and domain level.**


PREFIX sio:  <http://semanticscience.org/resource/>
PREFIX ado: <http://scai.fraunhofer.de/AlzheimerOntology#>
PREFIX rdfs:  <http://www.w3.org/2000/01/rdf-schema#>
PREFIX apgem: <http://www.apgem.org/resource/>
CONSTRUCT{
  # MCI value at formal level
  ?staging sio:SIO_000300 ?inferred.
  # MCI at domain level
  ?staging a ado:Mild_cognitive_Impairment.
}
WHERE {
     # Count T-Scores < 35
    {
      SELECT ?mmsetotal (COUNT(?tscore) AS ?tscorecount)
      WHERE {
        ?this sio:SIO_000062 ?csExperiment.
        ?csExperiment a apgem:apgem_0004 ; sio:SIO_000312 ?csdata.
        ?csdata sio:SIO_000028*/sio:SIO_001277 ?mmse.
        ?mmse rdfs:label “MMSE_Total”; sio:SIO_000300 ?mmsetota.
        ?csdata sio:SIO_000028*/sio:SIO_001277 ?score.
        ?score rdfs:label ?label ; sio:SIO_000300 ?tscore.
        # The variables must be Tscores 
        FILTER (regex(?label , “VOSP_Tscore”)
        || regex(?label, “CERAD_Recall_Tscore”) 
        || regex(?label, “COWAT_Tscore”) 
   
    || regex(?label, “TMTB_Tscore”)).
        FILTER (?tscore < 35)
    }
   group by ?this ?mmsetotal
 }
 ## data from Medical History experiment
 ?this sio:SIO_000062 ?mhExperiment.
 ?mhExperiment a apgem:apgem_0003 ; sio:SIO_000312 ?mhdata.
 # Participant informed subjective cognitive decline
 ?mhdata sio:SIO_000028*/sio:SIO_001277 ?cmhpar.
 ?cmhpar rdfs:label “P_subcogdec” ; sio:SIO_000300 ?P_subcogdec.
 # Informant informed subjective cognitive decline
 ?mhdata sio:SIO_000028*/sio:SIO_001277 ?cmhinf.
 ?cmhinf rdfs:label “I_subcogdec” ; sio:SIO_000300 ?I_subcogdec.
 # MCI Criteria
 FILTER(
   # 23 < MMSE 
   23 < ?mmsetotal)
   # Participant or informant cognitive decline 
   && (?P_subcogdec != 0 || ?I_subcogdec != 0)
   # One or more t-scores < 35 
   && ?tscorecount >= 1)
  # Diagnosis experiment to update
  ;?this sio:SIO_000062 ?diagExperiment.
  ?diagExperiment a apgem:apgem_0001 ; sio:SIO_000312 ?diagdata.
  ?diagdata sio:SIO_001277 ?stagnode.
  ?stagnode rdfs:label “stag”; sio:SIO_000300 ?staging.
  BIND(6 as ?inferred.
}

### Reporting and data extraction

XNAT provides various means to customize reports and searches to make them accessible through the web interface, such as the advanced use of display files. However, advanced XNAT displaying customization requires good knowledge of the underlying XNAT database structure (for customized SQL views and displays). Also its REST API enables the development of customized scripts. While this method is very powerful for external software development and library design (such as PyXNAT), it requires a fair amount of programming to perform complex queries and data retrieval.

Concept generalization (class subsumption in ontologies) and the graph-based model of RDF provide a powerful and flexible environment for query design. The use of ontologies and SPARQL for “intelligent querying” has been demonstrated many times in the literature (Pathak et al., 2012a,b; Leroux and Lefort, 2015) and is one of the inspirations for the development of our framework. It simplifies the creation of targeted reports and the extraction of subsets of data from different domains for further analysis. For instance, generating CSV files from SELECT clauses or RDF graphs with CONSTRUCT clauses.

Code 3 shows the query employed for tracking subjects that have Diffusion Tensor Imaging (DTI) and are diagnosed with MCI.

Code 3 **Query for tracking subjects that have Diffusion Tensor Imaging with a specific diagnosis staging.**


PREFIX sio: <http://semanticscience.org/resource/>
PREFIX rdfs: <http://www.w3.org/2000/01/rdf-schema#>
PREFIX apgem: <http://www.apgem.org/resource/>
PREFIX dicom: <http://purl.org/nidash/dicom#>
SELECT (count (?subject) as ?total)
WHERE {
  ?subject a sio:SIO_000399; sio:SIO_000062 ?session, ?diagnosis. 
  ?session a apgem:apgem_0028; sio:SIO_000312 ?sessiondata.
  ?sessiondata sio:SIO_000028*/sio:SIO_001277 ?desc.
  ?desc a dicom:seriesDescription; sio:SIO_000300 description.
  # staging information
    ?diagnosis a apgem:apgem_0001; sio:SIO_000312 ?diagdata. 
  ?diagdata sio:SIO_001277 ?stag. ?stag rdfs:label “stag;” sio:SIO_000300 ?stagValue.
  # Get only MCI staged subjects with labels starting with D10
  # with MRSessions with ID ending with _1 and have scans with
  # DTI in its series description 
  FILTER(?stagValue = 6 
    && regex(?subject, “^D10”) 
    && regex(?description, “DTI”)
    && regex(?expLabel, “-1$”))
}
GROUP BY ?subject

## Discussion

Comparing the framework to similar approaches is not straightforward, as the benefits are focused in improving development tasks and the assessment may be subjective, dependent on the objectives pursued. We have presented several use cases to illustrate the effectiveness and ease of use of the proposed solution.

The use of ontologies and semantic technologies as a means of data storage, access, and analysis is widely adopted in biomedical projects. However, this type of ventures still comprises a set of challenges. The most time consuming task of them has been the ontology selection, alignment, and mapping. Despite the great availability of different ontologies to the scientific community, many of them overlap in some subsets and/or lack some others, drawing a landscape of competing standards.

The selection of the technologies involved in the transformation, reasoning, and storing of the data is also up to discussion. It is important for the developers to evaluate and find a balance between ease of deployment and performance optimization, which will ultimately depend on the objectives pursued. Using query rewriting approaches like Ontop (Calvanese et al., 2015) saves development time, but at the expense of performance, which is bound to the complexity of the ontology and mappings. For instance, the rewriting of the queries suffers an exponential blow-up in the worst case (Gottlob et al., 2014). To overcome these problems, the complexity of the ontology needs to be restrained, which would potentially limit the flexibility of the ontological design. Also, the SQL source queries for the mappings need to be as optimal as possible. This task requires good knowledge of both SQL and XNAT database structure. Last but not least, the reasoning capabilities are also limited.

Regarding the use of the framework, the preliminary applications show promising results. QC is tightly integrated in the data update workflow, enabling the early detection of noisy and inconsistent data, saving a significant amount of time in data inspection. The data exposed in Fuseki's SPARQL endpoint allows data researchers to prepare very specific datasets in less time. As we thought, the preliminary results obtained by the stage classifier have highlighted discrepancies between its output and the actual diagnosis. Further analysis will be necessary to evaluate the source of these disagreements, which may be due to the simple approach of the current staging algorithm, errors in the data or in the diagnostic process. It opens the way for future applications of the framework.

While the implemented semantic environment already fulfills many of our motivations, there is still room for further improvements. One of the immediate enhancements for our framework is the alignment of the formal level with Linked Data Cubes to generate more self-contained datasets for external analysis. This is easily implemented with dedicated SPARQL constructs that translate from one vocabulary to another. The cubes and slices can be optimized to fit specific Machine Learning algorithms, saving intermediate adaptation steps. Another interesting use for the framework would be information retrieval and annotation of free text comments attached to many different experiments. The challenge mainly lies in the multilingual nature of the comments.

Although the development focuses on the XNAT platform, the modeling and techniques applied foster reutilization and are easily generalizable to other of the available archiving solutions for neuroimaging and clinical data. The only requirement would be the adaptation of the transformations and domain specific conceptualizations.

## Conclusion

We have presented an incremental, modular, and scalable framework that enhances and extends the capabilities of neuroimaging and biobanking systems through the use of semantic technologies. The approach has been exemplified through the XNAT platform in the context of the APGeM project.

The union of schemas, ontologies and services that together enable semantic data access composes the framework. XNAT model, along with XCEDE and complementary schemas, establish the schema level of the framework, providing a suitable means to consume and exchange imaging and clinical research data. The domain level provides the higher level with more abstract concepts, supporting simpler queries and knowledge modeling. The formal level, which works with low-level and raw data/metadata, provides a good toolset for Quality Control and consistency check. Integrating the reasoner in the pipeline allows taking advantage of the formal definitions, generating further assertions about data quality and classifications.

This work shows that following the proposed methodology is possible to enhance non-semantic biomedical research systems with semantic capabilities, improving data management from low-level data to more descriptive logical concepts. The use cases shown confirm the benefits of applying layered semantic descriptions to multi-dimensional datasets, common in the Neuroscience domain, highlighting the convenience of integrating these technologies in current systems updates and future developments.

## Author contributions

All authors participated in the conception, design and implementation of the work, and in the drafting and revision of the paper.

### Conflict of interest statement

The authors declare that the research was conducted in the absence of any commercial or financial relationships that could be construed as a potential conflict of interest.

## Footnotes

^1^https://www.w3.org/TR/owl2-primer/

^2^https://www.w3.org/TR/rdf11-primer/

^3^https://www.w3.org/TR/sparql11-overview/

^4^https://www.cdisc.org/standards/transport/odm

^5^http://www.neurodegenerationresearch.eu/

^6^http://www.neurodegenerationresearch.eu/publication/apgem/

^7^https://www.w3.org/TR/xmlschema-0

^8^https://www.w3.org/TR/prov-dm/

^9^http://ifomis.uni-saarland.de/bfo/

^10^http://nidm.nidash.org/

^11^http://obofoundry.org/ontology/pato.html

^12^https://loinc.org/

^13^https://www.w3.org/TR/xslt

^14^http://spinrdf.org/

^15^https://jena.apache.org/documentation/fuseki2

^16^https://joinup.ec.europa.eu/catalogue/distribution/study-persistent-uris-identification-best-practices-and-recommendations-topic
